# Spontaneous Fracture of the Fifth Metatarsal Secondary to Gout Tophus in a Young Patient: A Case Report

**DOI:** 10.7759/cureus.40400

**Published:** 2023-06-14

**Authors:** Ophélie Ménez, Nicolas De Saint Aubain, Riccardo De Angelis

**Affiliations:** 1 Radiology, Centre Hospitalier Universitaire (CHU) Saint-Pierre, Brussels, BEL; 2 Pathology, Institut Jules Bordet, Brussels, BEL; 3 Radiology, Institut Jules Bordet, Brussels, BEL

**Keywords:** ultrasound imaging, x-ray images, gout disease, mri images, fifth metatarsal fracture, tophaceous gout

## Abstract

Gout is a common disease, and its prevalence is increasing. After several years of untreated gout, in very rare cases tophi may cause a spontaneous fracture. This type of fracture may be difficult to distinguish from others, especially when gout is not yet diagnosed. We present a case of a pathological fracture caused by tophus in a young man, which led to the diagnosis of gout.

## Introduction

Gout is a common and treatable chronic metabolic disease caused by the deposition of monosodium urate microcrystals in joints and tissues related to chronic hyperuricemia. It manifests as intermittent episodes of very painful arthritis (gout flares) caused by an immune system response to monosodium urate crystal deposits [1].

The succession of gouty flares not adequately treated can lead to the development of gouty tophi. In very rare cases, these can lead to bone fragility resulting in fractures of the underlying bone. This complication is rarely described, despite the increasing frequency of the disease [2]. We report a case of a young patient for whom a pathological fracture on a tophus allowed the diagnosis of gout.

## Case presentation

A 30-year-old male presented himself to the emergency department with spontaneously occurring pain around the lateral border of his left foot that started one week earlier. The patient has no history of trauma, oncologic or inflammatory disease. At clinical examination, soft tissues were edematous in the painful region, and limb movements were limited. No other region presented with pain. Blood tests were overall normal, aside from a light inflammatory syndrome.

**Figure 1:**
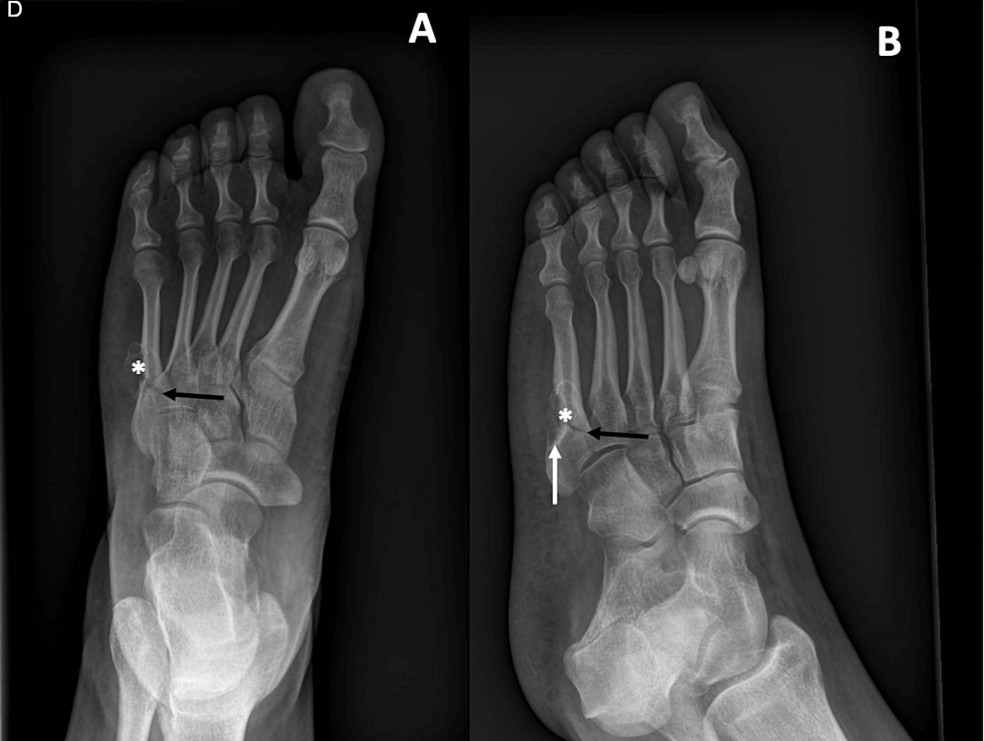
Radiographical findings Radiographs of the right foot revealed a transverse fracture of the fifth metatarsal (black arrow), a soft tissue lesion (white star), and bone erosions of the facing cortex (white arrow).

**Figure 2:**
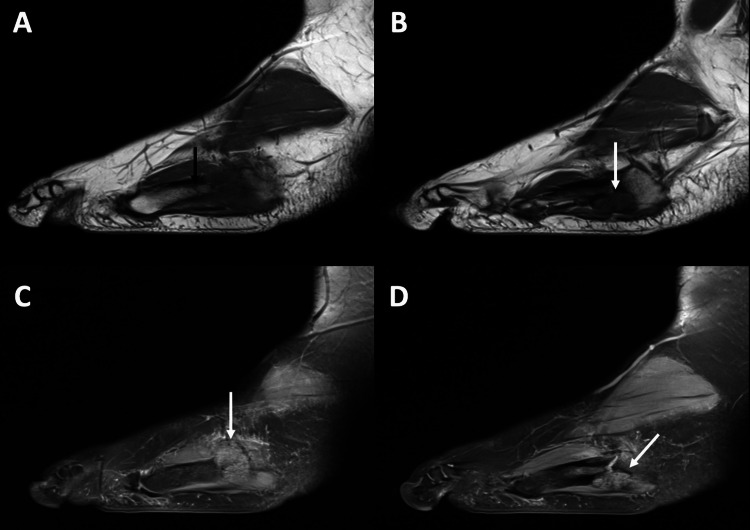
Magnetic resonance imaging findings Magnetic resonance imaging (MRI) showed the fracture (A) (black arrow) and a nodular lesion (white arrow), with hyposignal on sagittal T1 (B), and iso-signal on sagittal T2 DP (C,D).

**Figure 3:**
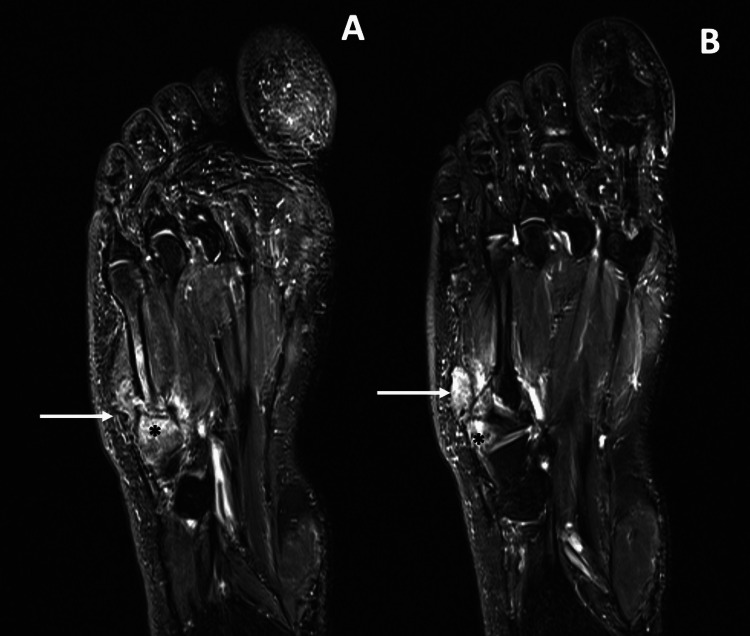
Contrast-enhanced magnetic resonance imaging findings Gadolinium-enhanced MRI showed intense enhancement of the nodular lesion (white arrow) and adjacent bone osteolysis (black star).

An X-ray was performed and revealed a non-displaced transverse fracture of the proximal shaft of the fifth metatarsal bone (Figure 1). Also, in adjacent soft tissues, a rounded-shaped lesion with a sclerotic edge was detected. Cortical erosion was also present. A complementary MRI was performed (Figure 2). It confirmed an 18mm rounded-shaped lesion in the soft tissues adjacent to the metatarsal fracture, with osteolysis of the cortex. This lesion presented an intermediate signal intensity on the T2-weighted images and decreased signal intensity on the T1-weighted images. Also, it presented a peripheral enhancement after gadolinium injection (Figure 3). Other nodular lesions, with similar characteristics to the first, were located on the plantar aspect of the base of the second and third metatarsals.

This clinical and radiological presentation led to the initial suspicion of a pathological fracture of neoplastic origin. However, multifocal involvement suggested a diagnosis of tophi. A biopsy was therefore suggested to exclude a neoplastic origin.

**Figure 4:**
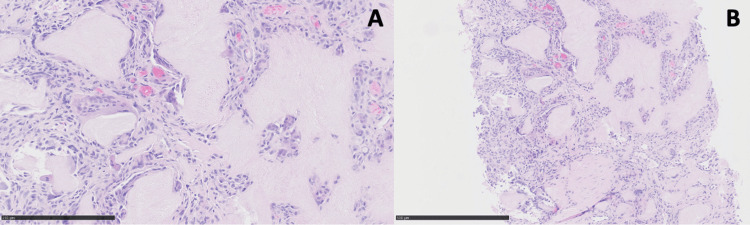
Microscopic analysis findings Microscopic analysis showing filamentous materiel associated with a giant cell granulomatous reaction. Enlarged section (A: x20, B: x10).

An ultrasound-guided biopsy was performed. The specimen showed filamentous material associated with a granulomatous giant cell reaction, confirming the diagnosis of gouty tophus (Figure 4).

The patient was treated effectively with non-steroidal anti-inflammatory drugs (NSAIDs), colchicine, and immobilization and is currently monitored for tophaceous gout.

## Discussion

Gout is a chronic disease with an increasing incidence (incidence ranges between 0·6 and 2·9 per 1000 person-years), probably due to the aging of the population and changes in lifestyle, notably with an increase in the number of metabolic syndromes and associated pathologies. Usually, a chronic hyperuricemia background is present and may be asymptomatic for several years before the first gout flares. In more than half of the cases, the first gout attack is a monoarthritis of the first metatarsophalangeal joint [1,3]. The recurrent nature of the attacks, the duration, and location of the arthritis, as well as biological and radiological elements, generally allow the diagnosis to be made. After several years of untreated gout, tissue accumulation of uric acid forms tophus [1]. In rare cases, the presence of a tophus is the first sign of gout [3].

Gouty tophi are frequently complicated by pain, reduced mobility, or ulceration [1]. Pathological fractures at the site of a tophus are a rare complication of gout, with only a few cases described in the literature [2,4]. In the case presented in this article, the fracture led to the diagnosis of gout, which is particularly rare [2].

In 2010, Nguyen et al. reviewed a total of 13 cases of fracture in tophaceous gout. The most common site for this type of fracture is the patella, with only one case located on the fifth metatarsal [2]. A second case of fracture is secondary to gout localized to the fifth metatarsal was described in 2011, with no associated tophus [4]. Gout, in general, does not appear to be a risk factor for fracture [5], but tophaceous gout is characterized by an increased development of osteoclasts within tophi [2].

Non-invasive medical imaging is an essential tool in the diagnosis of gout, mainly through ultrasound and dual-energy computed tomography. Conventional radiographs are more useful in advanced stages, where they allow us to observe bone erosions but also rarer complications such as fractures, as in our case. When doubt persists, the presence of monosodium urate microcrystals in synovial fluid or tophi allows the diagnosis of certainty [6,7].

## Conclusions

Spontaneous fractures caused by gouty tophi are rare and difficult to diagnose, especially if the context of gout is not known, as in our case. Correct knowledge of imaging and clinical features can usually help distinguish this type of pathological fracture from others.
